# Impact of gestational stress on bone and body composition in Wistar rats after lactation

**DOI:** 10.1590/1414-431X2025e14551

**Published:** 2025-10-06

**Authors:** M.A.C. Martins, J.A.S. Monnerat, G.F. Teixeira, B.B. Lucchetti, J. Mentzinger, L.L. Velasco, F.A. Sodré, E.M. Silva, H.N.M. Rocha, A.C.L. Nóbrega, N.G. Rocha, G.S. Rocha, R.F. Medeiros

**Affiliations:** 1 Laboratório de Nutrição e Metabolismo, Departamento de Nutrição e Dietética, Universidade Federal Fluminense, Niterói, RJ, Brasil; 2 Laboratório de Cardiometabolologia Integrativa, Departamento de Fisiologia e Farmacologia, Universidade Federal Fluminense, Niterói, RJ, Brasil; 3 Laboratório de Ciências do Exercício, Departamento de Fisiologia e Farmacologia, Universidade Federal Fluminense, Niterói, RJ, Brasil; 4 Laboratório Analítico de Biomateriais Restauradores, Departamento de Odontologia Restauradora, Universidade Federal Fluminense, Niterói, RJ, Brasil

**Keywords:** Maternal stress, Variable stress, Bone health, Pregnancy, Body composition

## Abstract

This research aimed to evaluate the impact of stress at the end of the pregnancy on bone health and body composition of Wistar rats after the breastfeeding period. The 90-day pregnant Wistar rats were divided into 2 groups (n=7/group): the stress group (SG) and the control group (CG). The stress protocol was carried out over 8 days, with 4 different types of stressor stimuli during the third week of pregnancy. Serum corticosterone, osteoprotegerin (OPG), nuclear factor kappa beta ligand (RANKL), calcium, and phosphorus were measured by an ELISA kit and body composition by the carcass technique. Bone mineral density, content, and area were measured in the femur by dual-energy X-ray absorptiometry, and the maximum force, breaking force, and elastic modulus were measured using a flexural test with a load cell (50 kgf). Results with P<0.05 were considered significant and data are reported as means±SD. Corticosterone was higher in SG (P=0.04), showing the effectiveness of the protocol, but no differences were observed in OPG, RANKL, calcium, phosphorus, and body composition. SG had lower bone mineral density (P=0.01) and lower maximum strength (P=0.04). Stress during the gestational period promoted deleterious effects on maternal bone health after the lactation period, shown by the reduction of bone mineral density and maximum strength, affecting bone quality; no difference was found in body composition.

## Introduction

Mental health can be affected by stressful events that are linked to anxiety disorders and depression symptoms. About 20-25% of people who experience long periods of intense stress develop depression (1). Stress is defined as a threat to the body's homeostasis with the activation of the sympathetic nervous system (SNS) and the hypothalamic-pituitary-adrenal axis (HPA), resulting in the release of catecholamines, glucagon, and corticosteroids (2). Prolonged exposure to stress, whether environmental, physiological, or psychological (2-5), leads to negative consequences and increases the susceptibility to cardiovascular diseases, cancer, and osteoporosis (1).

During pregnancy, several maternal physiological changes occur in the mother that can affect fetal development and lactation and increase the mother's susceptibility to the harmful effects of stress (6). Studies have already pointed out some effects of gestational stress, such as structural malformations, low birth weight, and altered body composition in the fetus as well as increased risk of gestational diabetes, pre-eclampsia, metabolic disorders, and bleeding in the mother (7-11).

Furthermore, hormonal changes during pregnancy and lactation affect both intestinal calcium absorption and bone formation and resorption, which may characterize bone remodeling. This remodeling is regulated by the receptor activator of nuclear factor kappa B (RANK), RANK ligand (RANKL), and osteoprotegerin (OPG) axis. RANKL is an osteoclastogenic cytokine produced by osteoblast precursor cells, which binds to RANK on osteoclast precursor cells. The RANK-RANKL binding stimulates the differentiation of osteoclast precursors into pre-osteoclasts, which fuse and form osteoclasts. Osteoblasts also secrete OPG, which binds to RANKL and inhibits RANK-RANKL binding, consequently decreasing osteoclastogenesis and bone resorption. Therefore, the RANKL and OPG rate is a reliable indicator of the rate of bone resorption (12,13). In this sense, exposure to a specific stress activates the HPA axis, leading to an increase in serum corticosterone, inhibiting bone formation by osteoblasts, and stimulating resorption by osteoclasts (14).

Bone demineralization also increases during lactation to supply minerals to the milk (15), with recovery only from 6 to 24 months after the end of lactation. Then, the skeletal system of the mother becomes more susceptible to stress in this interval. However, few studies have demonstrated the effects of stress on a woman's bone metabolism during critical periods of life, such as pregnancy or lactation. Therefore, the present study may help develop strategies for maintaining a woman's bone health after lactation.

Considering that exposure to stress during pregnancy may cause changes in the maternal skeletal system, the present study aimed to determine the impact of unpredictable stress at the end of the gestational period on bone health and body composition in *Wistar* female rats in the post-breastfeeding period.

## Material and Methods

The research was developed at the Laboratory of Exercise Sciences, Fluminense Federal University (UFF). The study was approved by the Ethics Committee on the Use of Animals (CEUA/UFF 9518170621).

### Experimental design

Wistar rats from the Laboratory Animal Center at UFF were used. During the entire experimental period, the animals were kept in polypropylene cages in the Biomedical Institute, under a temperature-controlled environment (24±2°C), adequate lighting (12-h light/dark cycle), standard commercial food (Nuvilab^®^, Brazil), and water *ad libitum*.

When the adult rats were 90 days old, with body mass between 230 and 250 g, they were mated in collective polypropylene cages, in a proportion of two females to each male. After confirmation of pregnancy (T0), confirmed by the vaginal wash technique or the presence of a tampon (16), rats were randomly allocated into control group (CG) and stress group (SG) (n=7/group), and placed into individual cages until the offspring birth.

The SG rats were subjected to the daily unpredictable stress protocol (Table 1) from the 14th to the 21st gestational day (T14-T21, equivalent to the 3rd trimester of pregnancy in humans). The mothers were euthanized 30 days after offspring birth (T51).

**Table 1:** Unpredictable stress protocol used in the study.

Day	Type of stress
T14	Isolation for 60 min
T15	Wet sawdust for 24 h (250 mL of tap water at room temperature)
T16	Cage tilt (5 cm) for 24 h
T17	Deprivation of food and water for 6 h (from 3 pm to 9 pm)
T18	Isolation for 60 min
T19	Wet sawdust for 24 h (250 mL of tap water at room temperature)
T20	Cage tilt (5 cm) for 24 h
T21	Deprivation of food and water for 6 h (from 3 pm to 9 pm)

T14-T21: days of the third week of pregnancy.

### Stress protocol

The rats from SG were subjected to the stress protocol during the third week of rat pregnancy (T14-T21), which consisted of 4 different stressful stimuli, one a day for 8 consecutive days, repeated twice following the order in Table 1 (17,18).

### Nutritional analyses

Twice a week until the end of the experiment, body mass and food consumption (FC) were monitored on a precision digital scale (Bel Engineering S3102, Italy). The food consumption of each animal was determined by subtracting the weight of the food remaining in the cage from the amount of food placed initially.

### Euthanasia and blood and tissue collection

Euthanasia was performed at the end of the experiment (T51). The animals were anesthetized with an intraperitoneal mento-pubic injection of xylazine (10 mg/kg) (Virbac Laboratory S.A.^®^, Brazil) and ketamine (80 mg/kg) (Virbac Laboratory S.A.^®^), with subsequent opening of the thoracic and abdominal cavities. They were euthanized by exsanguination through cardiac puncture. The collected blood was placed in a tube without anticoagulant and centrifuged at 3,500 *g*, 4°C for 15 min to obtain serum (Sigma^®^ 4K15 centrifuge, USA), then plasma was separated into microtubes (300 µL), frozen in liquid nitrogen, and stored in an ultrafreezer (-80°C) for subsequent biochemical analyses.

The right paw was collected, identified, and stored in a freezer at -20°C for bone analyses. The carcass of each animal was eviscerated, weighed on a digital scale, identified, individually packaged, and frozen at -20°C to assess body composition.

### Biochemical analyses

Serum levels of corticosterone (Cayman^©^, USA), OPG, and RANKL were evaluated using enzyme-linked immunosorbent assay kits, according to the instructions of the manufacturer (Finetest^©^, China). Calcium and phosphorus levels were evaluated using end-point colorimetric assays, according to the manufacturer's instructions (Bioclin^©^, Brazil).

### Body composition

Body composition was assessed using the carcass method, with quantification of protein and lipid content, as described by Stansbie et al. (19) and Cavalcante et al. (20), respectively.

### Bone analyses

The right femur was used for bone analyses. After thawing for 12 h at 8-10°C, the femur was separated from the tibia and other soft tissues, and subsequently analyzed, as previously described (21).

#### Bone size

The following measurements were carried out using a caliper with a reading accuracy of 0.01 mm: distance between the epiphyses (mm, distance between greater trochanter and lateral condyle), width of the midpoint of the diaphysis (mm), distance between greater trochanter and lesser trochanter (mm), and distance between lateral epicondyle and medial epicondyle (mm).

#### Dual-energy X-ray absorptiometry (DXA)

After drying the femur overnight at 25°C, the mass of the femur (g) was evaluated using an analytical balance (Shimadzu ATY224, Brazil). Bone mineral density (BMD; g/cm^2^), bone mineral content (BMC; g), and bone area (BA; cm^2^) were determined by DXA GE Lunar iDXA 200.368 (USA).

#### Biomechanical properties

Biomechanical properties were measured at three points using a flexural test on a universal testing machine (EMIC model 2000, Brazil), with a load cell of 50 kgf. The femur was placed on 2 supports 21.7 mm apart. Based on the force applied to the femur, the Instron software (IX series) generates a graphical tension load, from which the following properties were obtained: maximum force (MF; N), breaking force (BF; N), and elastic modulus (EM; Mpa).

### Statistical analyses

The results are reported as means±SD. Data were assessed for normality using the Shapiro-Wilk test. When data presented a normal distribution, the unpaired *t*-test was used. Mann-Whitney test was used when data were not normally distributed. P≤0.05 was considered significant. Statistical analyses were performed in the GraphPad Prism software version 8.0 (USA).

## Results

**Figure 1:**
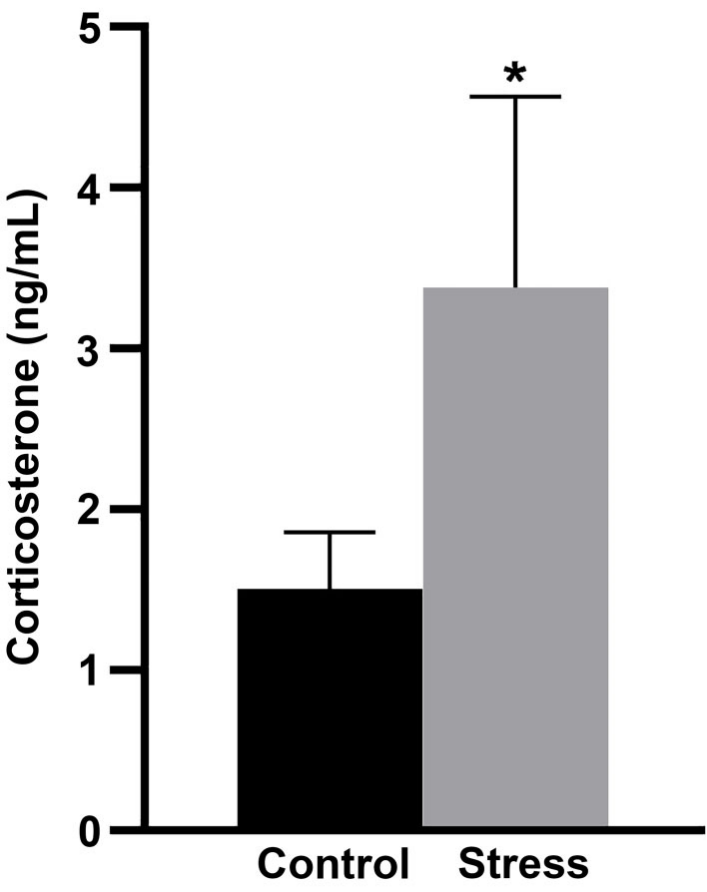
Serum corticosterone (ng/mL) from rats submitted to stress after lactation and control rats (n=7/group). Data are reported as means±SD. *P<0.05 (Mann-Whitney test).

Corticosterone levels (Figure 1) were significantly higher in SG (CG: 1.50±0.34 and SG: 3.37±1.18; P=0.04). There was no significant difference in body mass (CG: 258.4±10.06 g and SG: 257.4±8.04 g; P=0.84) and food intake (CG: 461.1±38.25 g and SG: 454.8±28.65 g; P=0.73) between the groups at the end of the experimental period.

There were no differences between groups regarding the levels of osteoprotegerin (GC: 150.9±46.89 pg/mL and SG: 128.8±32.59 pg/mL; P=0.22), RANKL (GC: 112.4±60.61 pg/mL and SG: 93.67±35.28 pg/mL; P=0.56), RANKL/OPG ratio (CG: 0.76±0.30 and SG:0.83±0.56; P=0.82), calcium (CG: 10.61±5.48 mg/dL and SG: 13.98±7.73, mg/dL; P=0.36), and phosphorus (GC:4.65±1.49 mg/dL and SG: 4.89±2.07 mg/dL; P=0.80).

As for body composition, no differences were observed between groups for protein (CG: 20.96±1.64% and SG: 20.28±3.92%; P=0.68) and lipid content (CG: 7.75±0.74% and SG: 8.31±1.15%; P=0.29).

**Figure 2:**
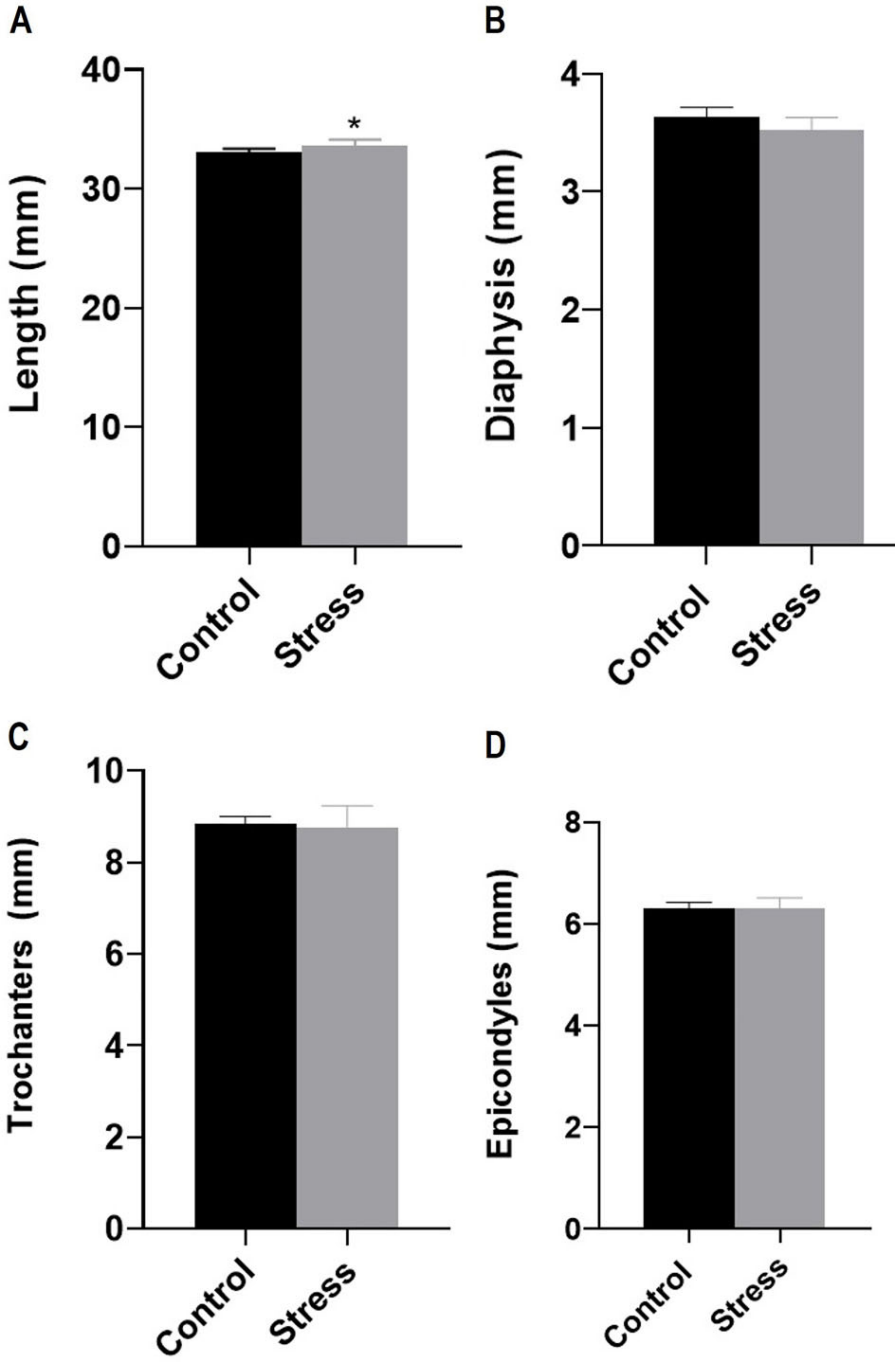
Bone parameters post-lactation of rats subjected to the daily unpredictable stress protocol from the 14th to the 21st gestational day and control animals. **A**, Femur length measured from the greater trochanter and the lateral condyle (mm); **B**, midpoint of the diaphysis (mm); **C**, distance between greater and lesser trochanters (mm); and **D**, distance between lateral and medial epicondyles (mm). Data are reported as means±SD (n=7/group). *P<0.05 (unpaired *t*-test).

Regarding bone parameters (Figure 2), femur length was greater in the SG compared to the CG (distance between epiphysis; CG: 33.05±0.29 mm and SG: 33.61±0.50 mm; P=0.02). However, no differences were observed in the midpoint of the diaphysis (CG: 3.63±0.08 mm and SG: 3.51±0.11 mm; P=0.06), distance between the greater and lesser trochanters (CG: 8.83±0.17 mm and SG: 8.77±0.47 mm; P=0.74), lateral and medial epicondyle (CG: 6.29±0.12 mm and SG: 6.28±0.22 mm; P=0.93), and femur weight between groups (CG: 0.629±0.02 g and SG: 0.6263±0.03 g; P=0.87).

**Figure 3:**
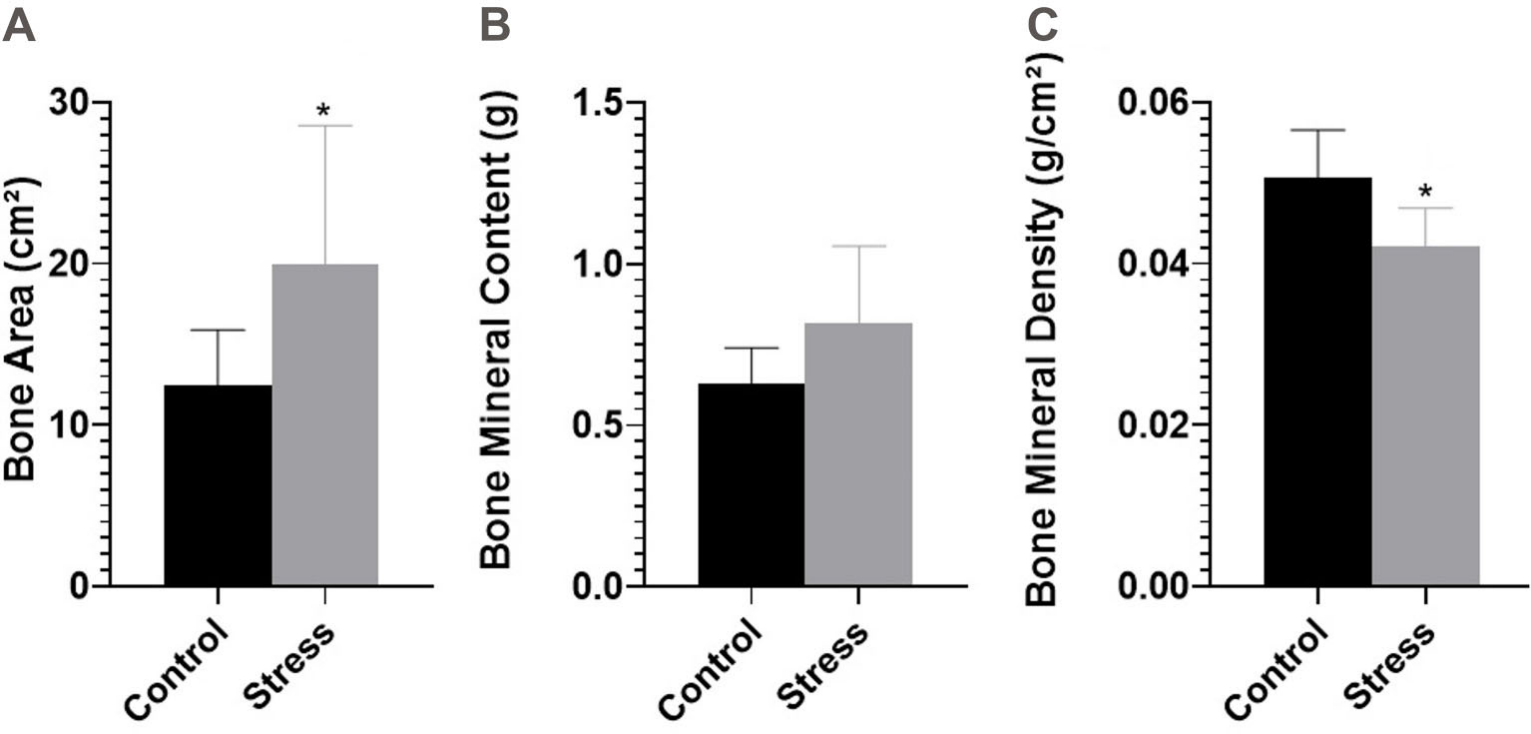
Bone characteristics post-lactation of rats subjected to the daily unpredictable stress protocol from the 14th to the 21st gestational day and control animals. **A**, Bone area (cm^2^); **B**, bone mineral content (g); and **C**, bone mineral density (g/cm^2^). Data are reported as means±SD (n=7/group). *P<0.05 (unpaired *t*-test).

Figure 3 demonstrates the DXA data. There was no significant difference in BMC (CG: 0.62±0.11 g and SG: 0.81±0.24 g; P=0.09). BA was greater (CG: 12.43±3.45 cm^2^ and SG: 20.00±8.56 cm^2^; P=0.05), whereas BMD was significantly lower (CG: 0.05±0.005 g/cm^2^ and SG: 0.04±0.004 g/cm^2^; P=0.01) in the SG compared to the CG.

**Figure 4:**
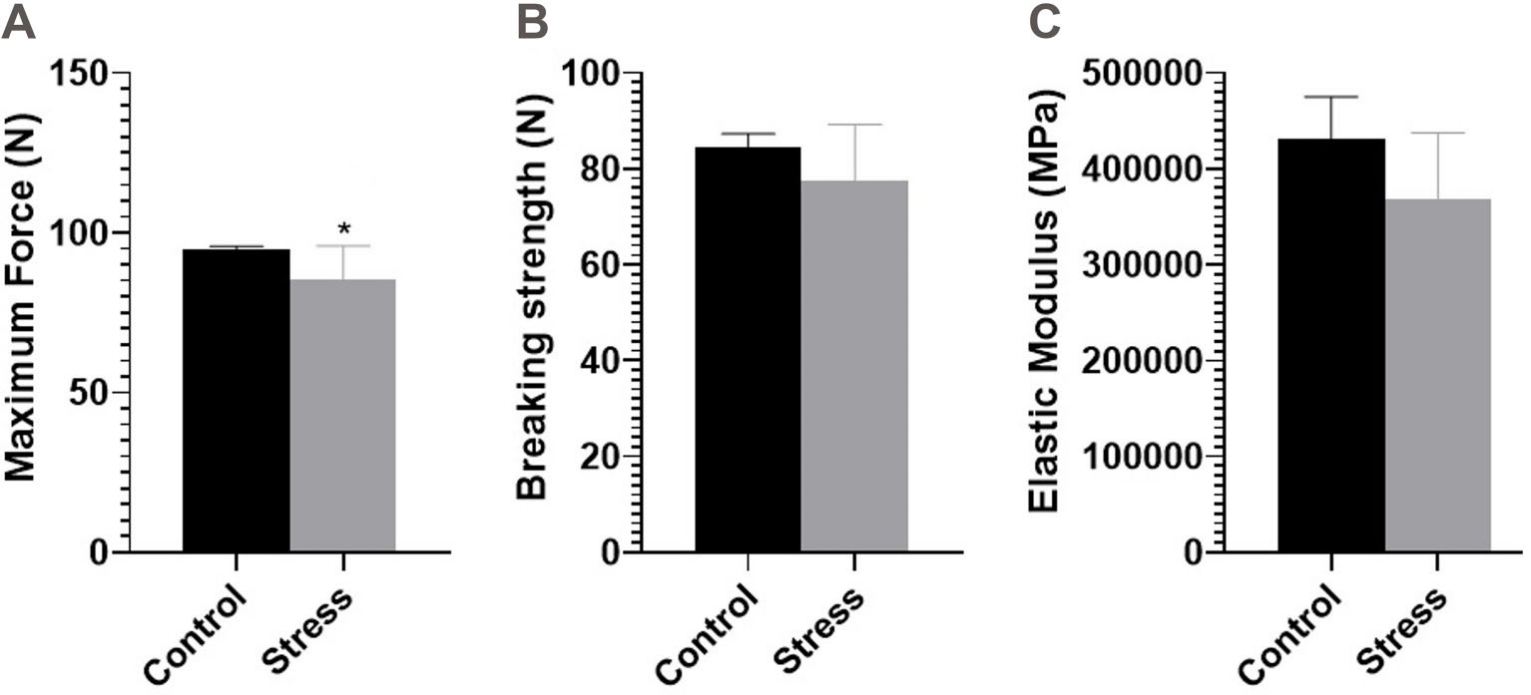
Measures of post-lactation bone strength of rats subjected to the daily unpredictable stress protocol from the 14th to the 21st gestational day and control animals. **A**, Maximum force (N); **B**, breaking strength (N); and **C**, elastic modulus (Mpa: Mega Pascal). Data are reported as means±SD (n=7/group). *P<0.05 (unpaired *t*-test).

Regarding biomechanical properties (Figure 4), the maximum force was significantly lower in the SG than in the CG (CG: 94.57±1.13 N and SG: 85.57±10.28 N; P=0.04). There were no differences in breaking force (CG: 84.57±2.69 N and SG: 77.43±11.84 N; P=0.14) and elastic modulus (CG: 430580±44710 MPa and SG: 369184±68588 MPa; P=0.07) between groups.

## Discussion

The present study investigated whether exposure to an unpredictable stress protocol during the third week of pregnancy in *Wistar* rats could lead to negative effects on bone and body composition after the lactation period. The main findings were higher corticosterone, lower BMD, and lower maximum force in the stress group.

Pregnancy, lactation, and the post-lactation period are important phases for the maternal skeletal system and metabolism due to the endocrine and morphological changes that occur in the woman's organism. Therefore, stressful events in those critical periods may induce overactivity of the HPA axis and in the nervous, cardiovascular and immune systems, leading to development or progression of diseases (22,23). The unpredictable stress protocol performed during pregnancy was able to increase post-lactation corticosterone levels, demonstrating the effectiveness of the protocol in activating the HPA axis even after the end of the stress protocol.

The literature shows that distinct stressful protocols can influence the rat's body composition. Artiga et al. (24) demonstrated that a 5-day shock stress protocol reduced fat mass in male animals. Similarly, Tamashiro et. al. (25) observed that chronic social stress decreased total body weight, fat, and lean masses during the experimental protocol, whereas it increased the fat mass after the end of stress. Paternain et. al. (26) also demonstrated that unpredictable stress carried out during the last week of pregnancy (14-21 days) reduced body mass, without alterations in food consumption in *Wistar* rats. In the present study, no significant differences in body composition were observed, which may be due to the assessment being carried out in the post-lactation period or the unpredictable stress protocol from the present study not evolving long periods of food deprivation.

Stress can lead to hyperprolactinemia during lactation (27), which is associated with lower levels of steroid hormones, dysfunctional bone metabolism, and consequently deleterious effects on the skeletal system (28). Studies have shown the relationship between hyperprolactinemia and hypogonadism with a greater chance of fracture and lower BMD (29).

Stress is also associated with decreased bone quality and loss of bone mass, possibly through hyperactivity of the SNS and the HPA axis, stimulating the release of corticotropin-releasing hormone, adrenocorticotropic hormone, and glucocorticoids, while decreasing gonadal and growth hormones (23). In addition, these stress-response mechanisms increase levels of interleukin-6, resulting in impairment of osteoblast metabolism and consequently leading to loss of bone mass (23).

In this study, the group submitted to the stress protocol presented higher levels of corticosterone, lower BMD, and lower maximum femur strength. Furthermore, a tendency of reduction in the elastic modulus was observed compared to the CG, showing lower bone quality and greater bone fragility. These results corroborated other studies in the literature that show that exposure to stress, whether in humans or animals, triggers harmful responses in the body, and consequently leads to loss of bone quality (30-32).

It is believed that serum corticosterone, released in response to stress, upregulates RANKL expression and downregulates OPG expression in osteoblasts, leading to greater RANK-RANKL binding and lower RANKL-OPG binding, increasing the osteoclastogenic activity and bone reabsorption (33). Although the bone damage caused by the stress protocol was evident, no significant differences were found in the serum levels of OPG and RANKL, as well as in the RANKL/OPG ratio, possibly because they were measured after lactation, indicating that there may have been a natural regulation of bone metabolism. On the other hand, bone quality was affected, suggesting that even with normalization or metabolic regulation, it was still not possible to reverse the damage from gestational stress. Macari et. al. (34) showed that there is a relationship between lactation and the RANK/RANKL/OPG axis by measuring RANK, RANKL, OPG, and prolactin in nulliparous lactating and non-lactating mice, indicating that all of them are involved in bone remodeling induced by lactation.

Serum levels of calcium and phosphorus can be influenced by some pathological conditions, hormonal levels, especially vitamin D and parathormone, or intense osteoblastic/osteoclastic activities (35). The literature is inconclusive regarding stress effects on serum calcium levels. While Morimoto et al. (36) showed that serum calcium levels decreased after a short or long restraint stress protocol, Fujimoto et al. (37) demonstrated that a water immersion stress protocol was not able to alter serum calcium levels, but serum calcitonin levels were reduced. These results showed that the type of stress, duration, damage caused, and time of measurement are fundamental for the variability of results.

This is the first study on the association between maternal stress suffered during pregnancy and bone damage in the post-lactation period and opens a new perspective for further investigations. It is not known whether the damage in the group that underwent the stress protocol could be reversed after lactation, as the body naturally recovers the bone mass lost during lactation. More studies on the subject are necessary to understand the repercussions on mothers' skeletal system.

Additionally, this study should be viewed under some limitations such as the lack of histological analyses to better understand the possible mechanisms related to damage caused by stress in the femur, as well as the lack of a study at different stages of pregnancy (gestation, lactation, and post-lactation) to observe the extent of bone damage and changes in resorption caused by stress in mothers. Furthermore, no behavioral analyses were conducted immediately after stress to verify the *in vivo* effect of the stress protocol, although the effectiveness of the protocol was confirmed through the measurement of plasma corticosterone.

Stress during pregnancy promoted deleterious effects on bone that lasted after the lactation period, which was indicated by the reduction of BMD and maximum bone strength, resulting in decreased bone quality.
